# Giant Aneurysm of the Left Main Coronary Artery: A Case Report

**Published:** 2018-01

**Authors:** Burak Açar, Sefa Ünal, Çağrı Yayla, Ahmet Göktuğ Ertem, Bihter Şentürk, Mustafa Özdemir, Mustafa Mücahit Balcı, Orhan Maden

**Affiliations:** 1 *Department of Cardiology, Yuksek Ihtisas Heart-Research and Education Hospital, Ankara, Turkey.*; 2 *Department of Radiology, Yuksek Ihtisas Heart-Research and Education Hospital, Ankara, Turkey.*

**Keywords:** *Coronary vessel anomalies*, *Coronary aneurysm*, *Multidetector computed tomography*

## Abstract

Coronary artery aneurysms are rare findings in patients referred for coronary angiography. Their prevalence ranges between 0.2% and 6% in different case series. We describe a male patient with a huge left main coronary artery aneurysm causing exertional angina, which was diagnosed with coronary angiography. All of the left coronary system arose from the aneurysm. He underwent coronary angiography again, followed by multislice computed tomography with a three-dimensional reconstruction. Although there is no known standard treatment modality for such aneurysms, we planned medical therapy after consultation with the cardiovascular surgery department. The patient’s first visit was in March 2013, and he was thereafter followed up until September 2016.

## Introduction

Coronary artery aneurysms are localized dilatations exceeding the diameter of the adjacent normal coronary segments. They are characterized by the abnormal dilatation of the vessel lumen, defined as a lumen with a diameter greater than 1.5-fold the diameter of the lumens of the normal vessels adjacent to it or the largest coronary vessel lumen.^   1 ^ The most commonly affected artery is the right coronary artery and, to a lesser extent, the proximal portion of the left anterior descending artery and the left circumflex artery, respectively.^   2 ^ These conditions constitute an important risk factor for the patient in terms of both morbidity and mortality. Atherosclerosis is the main cause of coronary anomalies in adults, whereas Kawasaki disease is the essential cause of these anomalies in children and adolescents. Other causes include trauma, polyarteritis nodosa, systemic lupus erythematosus, syphilis, and idiopathic.^   3 ^ Coronary artery aneurysms can give rise to many complications such as rupture, thrombosis, embolization, dissection, mechanical obstruction, and erosion to the adjacent structures.^   2 ^ The management of coronary artery aneurysms is controversial due to their rarity and unpredictable course. We herein describe a 53-year-old male patient with a giant left main coronary artery aneurysm.

## Case Report

**Figure 1:**
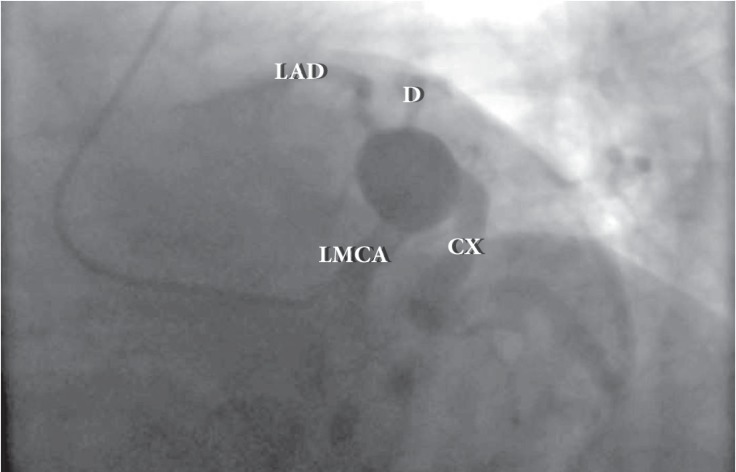
Left anterior oblique caudal view in the coronary angiogram, showing a giant left coronary aneurysm.

**Figure 2:**
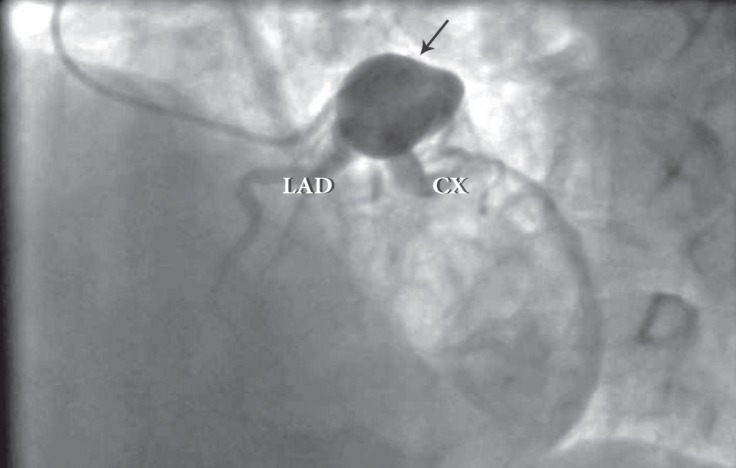
Left anterior oblique cranial view in the coronary angiogram, showing a giant left coronary aneurysm (arrow).

**Figure 3:**
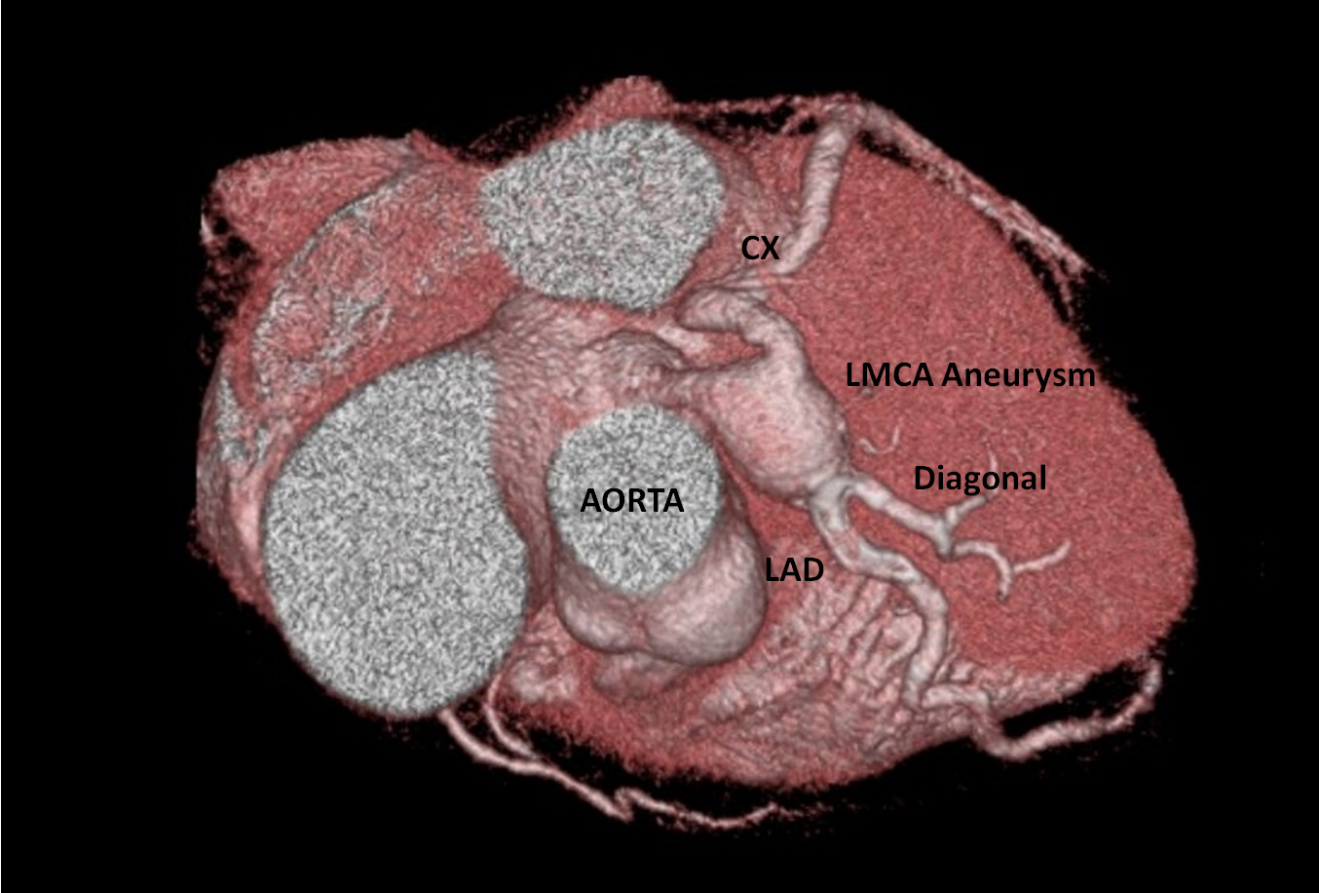
Three-dimensional volume-rendering computed tomography, showing a giant aneurysm of the left main coronary artery.

LMCA, Left main coronary artery; LAD, Left anterior descending artery; CX, Circumflex artery

**Figure 4:**
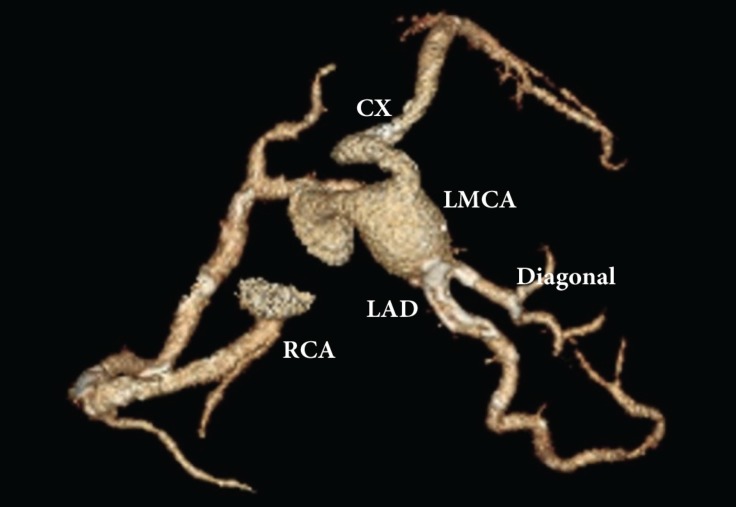
Another view of the giant aneurysm with three-dimensional volume-rendering computed tomography.

LMCA, Left main coronary artery; LAD, Left anterior descending artery; CX, Circumflex artery; RCA, Right coronary artery

A 53-year-old man was admitted to our cardiology department with exertional angina. He had hypertension and hyperlipidemia as cardiac risk factors. On physical examination, the patient’s vital signs were unremarkable and he had a New York Heart Association functional capacity of II. The respiratory and cardiovascular examinations were normal. The electrocardiogram showed nonspecific ST–T wave changes in leads V_1_–V_6_, and the laboratory work was within the normal range except for a low-density lipoprotein level of 170 mg/dL. The exercise stress test was nondiagnostic, and the myocardial perfusion scan revealed inducible ischemia in the anterior septum, mid, and basal portions of the anterior wall. Cardiac catheterization was performed and showed a giant left main coronary artery aneurysm (Figure 1 and Figure 2). All of the left coronary system arose from the aneurysm. The patient had no history of Kawasaki disease in childhood, and nor was the type of the lesion compatible with Kawasaki disease. Multidetector computed tomography coronary angiography confirmed a huge left main coronary artery, measuring 33 × 28 mm in size (Figure 3 and Figure 4). Because of the nonobstructive coronary artery disease in the other parts of the coronary system, the patient was managed with medical treatment. After 3 months of treatment, he referred to us again with exertional angina, which continued for more than 10 minutes. Coronary angiography illustrated no changes in his coronary system. After consultation with the cardiovascular surgery department, the patient was followed up with medical therapy. In the first hospitalization, medical therapy included acetylsalicylic acid (100 mg once a day), metoprolol (50 mg once a day), ramipril (5 mg once a day), isosorbide mononitrate (50 mg once a day), and trimetazidine HCL (35 mg twice a day). After the second hospitalization, warfarin treatment was added because of the giant aneurysm and slow flow. The patient’s first visit was in March 2013, and he was subsequently followed up until September 2016.

## Discussion

Coronary artery aneurysms are detected in 0.15% to 4.9% of patients undergoing coronary angiography.^   2 ^ The term “aneurysm” is defined as over 150% dilation of the largest diameter of the coronary artery and limited spherical or saccular dilation.^   2 ^ The most affected artery is the right coronary artery, followed by the left anterior descending and circumflex arteries.^   4 ^ The main cause of such anomalies is atherosclerosis in adults and Kawasaki disease in children.^   5 ^

 The standard treatment protocol has not been established for giant coronary aneurysms.^   6 ^ Such aneurysms might cause thromboembolism, rupture, or mass effect on the other structures. In the literature, there is no reported consensus about the treatment of huge aneurysms of the coronary arteries. The management options of these patients include percutaneous stenting with covered stents, medical management with antiplatelet therapy, and surgical therapy.^1^ Surgery or coil embolization is another treatment modality that can be used in this case. Tuncer et al.^1^ reported a case with a 3.0 × 2.0 cm saccular aneurysm, which was treated with medical therapy and at the 8 months’ follow-up, the patient was free of angina. Another patient with a giant left main coronary artery aneurysm, measuring 2.1 × 2.4 cm in size, was managed with surgery.^3^ The surgical techniques include isolating or resecting the aneurysm and reconstructing the coronary course by using an interpositional graft or by maintaining the distal coronary flow via concomitant coronary artery bypass grafting or performing thrombectomy.^   7 ^ With respect to our patient, we thought that the aneurysm was huge. In general, the thrombotic mechanism in these lesions occurs due to stasis such as venous thrombosis. Following detailed consultation, we opted to prescribe warfarin as medical therapy.

## Conclusion

The treatment of giant coronary artery aneurysms is still a subject of debate. A case-based approach should be drawn upon according to the patient’s symptoms, comorbid condition, and complexity of the lesions. 

## Notes:

***This paper should be cited as: ***Açar B, Ünal S, Yayla Ç, Ertem AG, Şentürk B, Ozdemir M, Balcı MM, Maden O. Giant Aneurysm of the Left Main Coronary Artery: A Case Report. J Teh Univ Heart Ctr 2018;13(1) 35-37.
